# Rationale and Design of the ADIDAS Study: Association Between Dapagliflozin-Induced Improvement and Anemia in Heart Failure Patients

**DOI:** 10.1007/s10557-021-07176-0

**Published:** 2021-03-29

**Authors:** Jianping Zeng, Yunlong Zhu, Wenjiao Zhao, Mingxing Wu, Haobo Huang, He Huang, ChunFeng Wu, XiaoLin Zhou, ShengHua Zhou, ChengMing Wang, Kai Yin, FangHua Xu, ZhiQiang Cai, Xinyang Li, Huiheng Cheng, Youwen Xie, Zhuqing Tan, Xiaoyong Hu, Dexiang Liao, Yingchun Wang

**Affiliations:** 1 Department of Cardiology, Xiangtan Central Hospital, Xiangtan, Hunan 411100 People’s Republic of China; 2 Department of Cardiology, The Second Xiangya Hospital of Central South University, Changsha, Hunan 410011 People’s Republic of China; 3 Department of Cardiology, Zhuzhou Central Hospital, Zhuzhou, Hunan 412000 People’s Republic of China; 4 Department of Cardiology, The First People’s Hospital of Xiangtan City, Xiangtan, Hunan 411100 People’s Republic of China; 5 Department of Cardiology, The People’s Hospital of Xiangtan County, Xiangtan, Hunan 411100 People’s Republic of China; 6 Department of Cardiology, Xiangxiang People’s Hospital, Xiangtan, Hunan 411400 People’s Republic of China; 7 Department of Cardiology, The Second People’s Hospital of Xiangxiang, Xiangtan, Hunan 411400 People’s Republic of China; 8 Department of Cardiology, JiangNan Hospital in Xiangtan, Xiangtan, Hunan 411100 People’s Republic of China; 9 Department of Cardiology, Shaoshan People’s Hospital, Xiangtan, Hunan 411300 People’s Republic of China; 10 Department of Cardiology, The Third People’s Hospital of Xiangtan, Xiangtan, Hunan 411100 People’s Republic of China; 11 Department of Cardiology, The First People’s Hospital of Zhuzhou County, Zhuzhou, Hunan 412000 People’s Republic of China; 12 Department of Cardiology, The Second People’s Hospital of Xiangtan, Xiangtan, Hunan 411100 People’s Republic of China

**Keywords:** Heart failure, Anemia, Dapagliflozin, Outcome

## Abstract

**Background:**

Heart failure (HF) is one of the most serious health concerns worldwide. Anemia is a highly prevalent comorbidity and outcome predictor in HF patients. Sodium glucose co-transport 2 (SGLT2) inhibitors have been demonstrated to reduce the risk of cardiovascular death and HF hospitalization in HF patients.

**Purpose:**

This investigator-initiated, interventional, prospective, double-blind, multicenter study is designed to investigate whether anemia correction is one of the prerequisites and determinants related to the beneficial effects of dapagliflozin in HF patients.

**Methods and Results:**

Up to 2030 HF participants receiving standard care will be randomly assigned to either oral dapagliflozin 10 mg once daily or placebo 10 mg once daily for 12 months. The primary outcome is the composite incidence of hospital admission for HF and all-cause death. Secondary outcomes include change in the Kansas City Cardiomyopathy Questionnaire (KCCQ) score and change in 6-min walk distance and hemoglobin level. Patients will be followed for 12 months after randomization.

**Conclusions:**

The ADIDAS trial offers an opportunity to assess the hemoglobin change and association between hemoglobin change and readmissions due to heart failure and all-cause death in patients with heart failure treated with dapagliflozin or placebo. This study could highlight if dynamic hemoglobin change is related to the outcome for HF patients.

**Trial Registration:**

ClinicalTrials.gov; NCT04707261. Registration date, 2020/12/01, “retrospectively registered”

## Introduction

Heart failure (HF) is a severe clinical syndrome with high mortality and rehospitalization rate. The morbidity of HF is 1.5–2.0% in developed countries, and the rate is even more than 10% in people over 70 years old [1]. According to the epidemiological survey in 2003, the prevalence of heart failure in Chinese adults aged 35–74 was 0.9% [2]. It is estimated that its incidence will significantly rise within the near future.

In the 2017 ACC/AHA/HFSA Focused Update of the 2013 ACCF/AHA Guideline for the Management of Heart Failure, anemia, hypertension, and sleep-disordered breathing were listed as important comorbidities of heart failure [3], among which the incidence of anemia was 33.3% and the incidence of anemia was similar between patients with preserved left ventricular ejection fraction (LVEF) with HF (HFpEF) and reduced LVEF with HF (HFrEF) [4]. Moreover, anemia is independently associated with HF disease severity [3], poor functional capacity [5], hospitalizations, and mortality [4]. Clinical trials have demonstrated intravenous iron for the treatment of anemia improve exercise capacity and quality of life (QoL), but did not affect overall mortality [6, 7].

Dapagliflozin is a sodium-glucose co-transporter 2 inhibitor (SGLT2i). Recent clinical trials showed that dapagliflozin use was associated with significantly reduced HF hospitalization [8], worsening heart failure or death from cardiovascular causes [9], and increased HF-related health status [10]. The Dapagliflozin Effect on Cardiovascular Events trial (DECLARE–TIMI 58) showed 27% reduction in HF hospitalization in the dapagliflozin group [8]. In the Dapagliflozin And Prevention of Adverse-Outcomes in Heart Failure trial (DAPA-HF), the incidence of composite outcome with worsening heart failure or death from cardiovascular causes was 16.3% in the dapagliflozin group versus 21.2% in the placebo group (hazard ratio, 0.74; 95% confidence interval (CI), 0.65 to 0.85; *P*<0.001) [9]. DEFINE-HF trial (Dapagliflozin Effects on Biomarkers, Symptoms and Functional Status in Patients with HF with Reduced Ejection Fraction) also demonstrated a meaningful improvement in HF-related health status or natriuretic peptides [10]. In another trial, treatment with dapagliflozin resulted in correction and prevention of anemia in patients with T2D, and hemoconcentration due to a diuretic effect (early phase) and increased erythropoiesis (late phase) might be the underlying mechanisms responsible for these effects [11]. Though clinical trials have demonstrated that SGLT2 inhibitors have impressive beneficial cardiovascular effects in HF patients both with and without diabetes, the actual mechanisms still remain elusive. Several potential theses have been proposed, including modulation on ventricular loading, cardiac energetics, sodium-hydrogen exchange, erythropoietin, progenitor cells, and CaMKII [12]. Studies also found that SGLT2 inhibitors could upregulate SIRT1, then activate hypoxia-inducible factor-2α (HIF-2α) and hypoxia-inducible factor-1α (HIF-1α), which can promote erythropoietin synthesis, and might contribute to erythropoiesis [13–16].

Anemia correction belongs to a feature of effective strategies on HF [17]; it remains unknown if anemia correction is a prerequisite of dapagliflozin-induced beneficial effects in HF patients. Thus, in this randomized clinical trial, we tested the hypothesis that anemia correction is a major determinant related to the beneficial effects of dapagliflozin in patients with heart failure.

## Methods

### Study Design

The primary objective of the ADIDAS trial (ClinicalTrials.gov Identifier: NCT04707261) is to investigate the hemoglobin change and the association between hemoglobin change and readmissions due to heart failure and all-cause death in patients with heart failure treated with dapagliflozin or placebo.

**Fig. 1:**
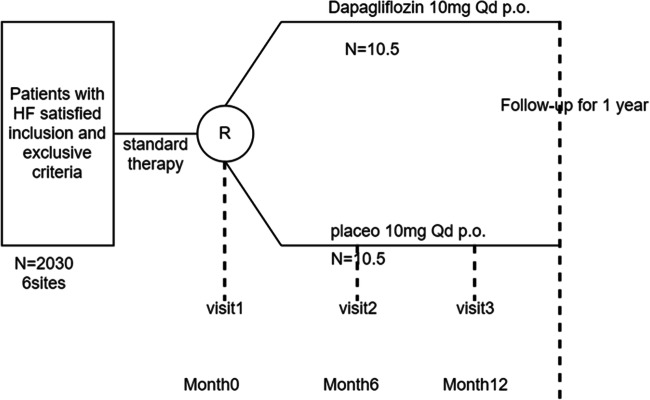
ADIDAS trial design diagram. R, randomization; Qd, once daily; p.o., orally

The study will include up to 12 sites within the Hunan Province, China, and up to 2030 participants receiving guideline recommended standard therapy will be computer-randomized to either oral dapagliflozin 10 mg once daily group or placebo 10 mg once daily group; the therapy duration is 12 months. Patients will be followed at 6 months and 1 year after randomization (Fig. 1).

### Participant Selection

Patients aged ≥18 years with elevated NT-proBNP or BNP levels on admission, ejection fraction (EF) <50%, and New York Heart Association (NYHA) class II, III, or IV are eligible for enrollment. Exclusion criteria include patients who received treatment with SGLT2i during the past 3 months, or previous intolerance of an SGLT2 inhibitor, or severe (eGFR<30 mL/min/1.73 m^2 by CKD-EPI), unstable, or rapidly progressing renal disease at the time of randomization, pregnant, and breastfeeding women (Table 1).

**Table 1:** Inclusion and exclusion criteria of the ADIDAS trial

Inclusion criteria ▪ Male or female between the ages of 18 and 100 years ▪ Elevated NT-proBNP or BNP levels on admission ▪ Ejection fraction of 50% or less, and New York Heart Association (NYHA) class II, III, or IV symptoms	
Exclusion criteria ▪ Treatment with SGLT2i during the past 3 months of admission, or previous intolerance of an SGLT2 inhibitor ▪ Severe (eGFR<30 mL/min/1.73 m^2 by CKD-EPI), unstable, or rapidly progressing renal disease at the time of randomization ▪ Pregnant or breastfeeding female patients	

### Endpoints

The primary endpoint is a composite of hospital admissions for HF and all-cause death. Secondary endpoints include change in the Kansas City Cardiomyopathy Questionnaire (KCCQ) score and change in 6-min walk distance as well as hemoglobin level.

### Randomization

Eligible patients will be computer-randomized in a 1:1 ratio to receive either oral dapagliflozin 10 mg once daily or placebo 10 mg once daily for 12 months.

### Study Visit and Follow-up

The study visit and follow-up are shown in Fig. 1. After obtaining informed consent, during visit 1, we check the inclusion and exclusion criteria and collect baseline information (including clinical examination and laboratory measurements). Patients are randomized to dapagliflozin or placebo, and study drug is dispensed. Visit 2 takes place 6 months and visit 3 12 months. At each visit, the patient’s well-being and the occurrence of adverse events (including hospitalization and all-cause death) are assessed. Patents are requested to complete the Kansas City Cardiomyopathy Questionnaire (KCCQ) and 6-min walk test, and blood sample is drawn and analyzed to check hemoglobin level.

### Sample Size Determination and Statistical Analysis

The ADIDAS study is powered on the event rates of previous large-scale study [9]. The incidence of composite outcome worsening heart failure or death from cardiovascular causes is 16.3% in the dapagliflozin group versus 21.2% in the placebo group (hazard ratio, 0.74; 95% confidence interval (CI), 0.65 to 0.85; *P*<0.001), as shown by DAPA-HF trial [9]. Thus, we estimated that with a total of 1990 patients (995 per group) required to determine the anemia correction-related beneficial effects on outcomes, the power of the study would be 80% with a 2-sided type I error rate of 0.05. Assuming that 2% of patients would withdraw or be lost to follow-up, the final sample size is determined to be 2030 patients (1015 per group).

All analyses will be detailed prior to database lock and unblinding. Summary tabulations will be presented for categorical variables, in which the number and percentage of participants within each category will be shown. As for continuous variables, the mean, median, standard deviation, first and third quartiles, and minimum and maximum values will be presented. The level of significance to be used for tests will be 0.05.

### Study Administration and Management

The trial is registered as ClinicalTrails.gov Identifier: NCT04707261. The local Institutional Review Board or Ethics Committee at each participating institution must approve the study, and all patients must provide written informed consent prior to enrollment. Funding is provided by the Department of Cardiology, The Xiangtan Central Hospital. The Xiangtan Central Hospital maintains the complete study database and will perform all key analyses. An independent data monitoring committee (DMC), composed of three physicians from the fields of cardiology and interventional cardiology and one biostatistician, reviews aggregate and individual patient data related to safety, data integrity, and overall conduct of the trial, on a periodic basis. The DMC may make recommendations to the steering committee and study sponsor as a result of its monitoring activities. The study has not started enrollment, and we plan to complete the enrollment of all subjects between February 2021 and January 2024, and the follow-up will last 1 year.

## Discussion

The ADIDAS trial is an investigator-initiated, interventional, prospective, double-blind, multicenter study involving patients with HF. All eligible participants receiving guideline recommended standard therapy will be computer-randomized to either oral dapagliflozin 10 mg once daily or placebo 10 mg once daily for 12 months.

The study is designed based on previous related clinical trials. The DECLARE–TIMI 58 trial [8] was a placebo-controlled, phase 3 trial in patients with type 2 diabetes who had or were at risk for atherosclerotic cardiovascular disease to receive either dapagliflozin or placebo; the result showed that dapagliflozin resulted in a lower rate of cardiovascular death or hospitalization for heart failure (4.9% vs. 5.8%; hazard ratio, 0.83; 95% CI, 0.73 to 0.95; *P*=0.005), which reflected a lower rate of hospitalization for heart failure (hazard ratio, 0.73; 95% CI, 0.61 to 0.88). The DAPA-HF study [9] demonstrated that dapagliflozin treatment lowed the risk of worsening heart failure or death from cardiovascular causes (16.3%) versus placebo treatment (21.2%) in HF patients, regardless of the presence or absence of type 2 diabetes. While DEFINE-HF trial demonstrated, there was a meaningful improvement in Kansas City Cardiomyopathy Questionnaire overall summary score or NT-proBNP, 61.5% of dapagliflozin-treated patients versus 50.4% with placebo (adjusted OR 1.8, 95% CI 1.03–3.06, nominal *P*=0.039) [10].

Though clinical trials have evidenced that dapagliflozin have impressive beneficial cardiovascular effects in HF patients both with and without diabetes, the actual mechanisms still remain elusive. Numerous theories have been proposed to explain the beneficial effects of SGLT2 inhibitors; one of them is the increase of erythropoietin (EPO) levels. Study showed that SGLT2 inhibitors could raise the hematocrit [18], even in those without diabetes (as seen in DAPA-HF); it is speculated that these agents may promote erythropoiesis via enhanced EPO secretion by the kidney [19]. Mazer et al. [19] recently explored the impact of empagliflozin on EPO levels, red blood cell (RBC) morphology, and indices of iron stores in individuals with type 2 diabetes (T2D) and stable coronary artery disease (CAD). It was shown that EPO levels increased significantly after 1 month of empagliflozin treatment, and hematocrit increased after 6 months by 2.34% [95% CI 1.1, 3.57, *P*<0.001]. Studies also showed that SGLT2 inhibitors can stimulate SIRT1 and then activate hypoxia-inducible factor-2α (HIF-2α) and hypoxia-inducible factor-1α (HIF-1α), which can promote erythropoietin synthesis [13–16]. In another trial, dapagliflozin corrected anemia in 52% of patients with anemia at baseline (placebo: 26%), and the mechanism may be a combination of hemoconcentration due to a diuretic effect (early phase) and increased erythropoiesis (late phase) [11].

Anemia is a common comorbidity in patients with heart failure and associated with more severe symptoms and an adverse prognosis [3, 20]. Recent study suggested that dapagliflozin has beneficial effects on HF, and this effect might be related to the correction of anemia. A recent trial showed that intravenous iron replacement with ferric carboxymaltose reduced the risk of heart failure hospitalizations in patients with iron deficiency after an episode of acute heart failure [21]. SGLT2 inhibitors possess multiple mechanisms of improving HF syndrome, including correction of anemia; and our study aims to determine if elevation of hemoglobin is definitely linked with improved outcome in HF patients receiving placebo or dapagliflozin, if dapagliflozin use is associated with higher incidence of elevation of hemoglobin, and last but not least, if elevation of hemoglobin is accompanied by HF outcome improvement.

## Data Availability

Data sharing is not applicable to this article now as patient enrollment is not started yet.
